# IL-17A-associated PTGS2 and MMP9 inflammatory signaling in ischemic stroke: clinical correlation and experimental evidence

**DOI:** 10.3389/fimmu.2026.1812571

**Published:** 2026-07-01

**Authors:** Chunlan Hou, Zilong Yang, Baoai Wang

**Affiliations:** 1 Graduate School, Shanxi Medical University, Taiyuan, Shanxi, China; 2 Department of Neurology, Fenyang Hospital of Shanxi Province, Fenyang, Shanxi, China

**Keywords:** IL-17A, ischemic stroke, MMP9, neuroinflammation, PTGS2

## Abstract

**Background:**

IL-17A is implicated in post-stroke inflammation, but its downstream inflammatory mediators in ischemic stroke remain incompletely defined. This study investigated whether IL-17A-associated signaling is linked to PTGS2 and MMP9 expression in ischemic stroke and whether IL-17A neutralization modulates these changes after cerebral ischemia.

**Methods:**

Three GEO datasets were analyzed to identify candidate genes and enriched pathways. Serum IL-17A, PTGS2, and MMP9 levels were measured by ELISA in patients with acute ischemic stroke and controls, and their associations with admission National Institutes of Health Stroke Scale (NIHSS) scores were examined. A rat middle cerebral artery occlusion (MCAO) model was established, and IL-17A neutralizing antibody treatment was used to assess effects on inflammatory markers, neurological deficits, and infarct volume.

**Results:**

Bioinformatics analysis prioritized PTGS2 and MMP9 as candidate inflammatory genes enriched in the IL-17 signaling pathway. Serum IL-17A, PTGS2, and MMP9 levels were significantly elevated in patients with ischemic stroke versus controls (all P < 0.001). IL-17A was positively correlated with PTGS2 (rho = 0.292, P < 0.01), MMP9 (rho = 0.216, P < 0.05), and admission NIHSS score. In MCAO rats, IL-17A, PTGS2, and MMP9 expression increased together with PGE2 and IL-6 levels. IL-17A neutralizing antibody treatment was associated with reduced PTGS2 and MMP9 expression, lower PGE2 and IL-6 levels, decreased infarct volume, and improved neurological deficit scores in MCAO rats, although an isotype-matched IgG control was not included.

**Conclusion:**

These findings support an IL-17A-associated PTGS2/MMP9 inflammatory network in ischemic stroke and suggest that IL-17A-associated inflammatory signaling may represent a biologically relevant pathway in post-ischemic injury, warranting further validation in studies incorporating appropriate isotype-matched antibody controls.

## 1 Introduction

Ischemic stroke (IS) is a leading cause of death and long-term disability worldwide (1). With ongoing population aging, the burden of stroke continues to rise, and outcomes are often poorer in older patients (2). The pathophysiology of ischemic stroke is complex, and post-ischemic neuroinflammation is widely considered an important contributor to secondary brain injury and impaired neurological recovery (3). Because aging is an important modifier of ischemic stroke susceptibility and post-ischemic injury, and aging-related biological processes may influence post-ischemic inflammation and neurovascular injury (4–6), CellAge was used as an exploratory resource to prioritize candidate genes with aging/senescence-related annotations.

Interleukin-17A (IL-17A) is a potent proinflammatory cytokine produced mainly by Th17 cells and γδ T cells (7). Previous studies have shown that IL-17A increases in injured brain tissue and peripheral blood after cerebral ischemia (8), and higher IL-17A levels have been associated with larger infarct volume and poorer clinical outcomes (9). However, the downstream inflammatory molecules linked to IL-17A signaling in ischemic stroke remain incompletely characterized, and their relevance to tissue injury after stroke requires further clarification.

To identify candidate inflammatory mediators potentially linked to IL-17A-associated signaling, we performed bioinformatics analysis and prioritized prostaglandin-endoperoxide synthase 2 (PTGS2/COX-2) and matrix metalloproteinase 9 (MMP9), two genes highly expressed in ischemic stroke datasets. PTGS2 is an inducible enzyme involved in arachidonic acid metabolism and prostaglandin synthesis (10), and its downstream product PGE2 is an important mediator of inflammatory responses (11, 12). MMP9 is a matrix-degrading protease involved in extracellular matrix remodeling and is commonly implicated in blood-brain barrier (BBB)-related injury after cerebral ischemia (13).

Pathway analysis suggested that PTGS2 and MMP9 were linked to IL-17-related inflammatory signaling. Given that the IL-17 family contains multiple ligands, including IL-17A, IL-17F, IL-17C, and IL-17E, we focused specifically on IL-17A because it is the IL-17 family cytokine most extensively studied in ischemic stroke and has the strongest existing evidence linking it to post-stroke neuroinflammation (14–16). In addition, IL-17A signaling through the IL-17RA/RC receptor complex is known to activate Act1-TRAF6-NF-κB/MAPK pathways (17–19), which are biologically compatible with induction of PTGS2 and MMP9 (20). Therefore, this study was designed as a hypothesis-driven analysis centered on IL-17A rather than a comprehensive comparison of all IL-17 family ligands. In this study, we combined bioinformatics analysis, clinical biomarker evaluation, and a rat middle cerebral artery occlusion (MCAO) ischemia-reperfusion model to examine whether IL-17A-associated inflammatory signaling is linked to PTGS2 and MMP9 expression in ischemic stroke and whether IL-17A neutralization modulates these changes *in vivo*.

## 2 Materials and methods

### 2.1 Bioinformatics analysis

We retrieved human ischemic stroke transcriptome datasets (GSE16561, GSE22255, GSE58294) from the GEO database (https://www.ncbi.nlm.nih.gov/geo/) (21). R language (v4.2.0) and limma package (22) were used to screen differentially expressed genes (DEGs) (screening criteria: |logFC| ≥ 0.585, FDR < 0.05). The CellAge database (https://genomics.senescence.info/cells/) was used as an exploratory gene-prioritization resource. The rationale for using this database was that ischemic stroke is strongly age-related, and aging/senescence-related biological processes may contribute to post-ischemic inflammation, vascular dysfunction, blood-brain barrier vulnerability, and impaired tissue repair. Therefore, we intersected the ischemic stroke-related DEGs with CellAge-annotated genes to prioritize candidates with both stroke-associated differential expression and prior annotation in aging/senescence-related biology. This step was not intended to define a cellular senescence signature or to screen inflammation-specific genes directly, but rather to narrow candidate genes within an age-related biological context for subsequent enrichment analysis and experimental evaluation.

To investigate the biological functions and the potential signaling pathways of these overlapping genes, gene ontology (GO) enrichment analysis and Kyoto Encyclopedia of Genes and Genomes (KEGG) enrichment analysis were performed using the R package “clusterProfiler” (version 4.12.6) (23). Gene set enrichment analysis (GSEA) was also performed using the R package “clusterProfiler”. These enrichment results were visualized using the “ggplot2” and “enrichplot” packages.

To explore the upstream regulatory mechanisms of the PTGS2 and MMP9, we performed transcription factor (TF) prediction analysis. TFs known to regulate both PTGS2 and MMP9 were retrieved from the TRRUST database (https://www.grnpedia.org/trrust/) (24). The resulting TF-gene regulatory network was constructed and visualized using Cytoscape software (version 3.10.2), where nodes represent TFs or genes and edges denote experimentally validated regulatory relationships.

### 2.2 Collection and detection of clinical samples

#### 2.2.1 Clinical participants

This study strictly adhered to the principles of the Declaration of Helsinki, and all clinical research protocols were approved by the Ethics Review Committee of Fenyang Hospital in Shanxi Province (approval number: 2025061). Written informed consent was obtained from all participants or their legal representatives. Patients with acute ischemic stroke and control subjects who visited the Fenyang Hospital in Shanxi Province from April 2025 to December 2025 were consecutively enrolled. The inclusion criteria for the ischemic stroke group were as follows: age ≥ 18 years; first-ever acute ischemic stroke; diagnosis confirmed by CT and/or MRI; admission within 24 hours after symptom onset; availability of blood samples and complete clinical data. The exclusion criteria were as follows: hemorrhagic stroke or transient ischemic attack; active infection at admission; autoimmune disease or other major inflammatory disease; malignant tumor; severe hepatic or renal dysfunction; recent surgery or major trauma; incomplete clinical data. For the control group, individuals with a history of stroke, active infection, autoimmune disease, malignant tumor, or other severe systemic diseases were excluded.

#### 2.2.2 Clinical data collection

Demographic and clinical data were collected from medical records, including age, sex, smoking history, alcohol consumption, diabetes, hypertension, leukoaraiosis, carotid plaque, admission National Institutes of Health Stroke Scale (NIHSS) score, and time from symptom onset to blood sampling, etc. Admission NIHSS score was used as an indicator of neurological deficit severity.

#### 2.2.3 Blood sample collection and biomarker measurement

Peripheral blood samples were collected from all participants at the time of enrollment. For patients with ischemic stroke, blood samples were obtained within 24 hours after symptom onset. After collection, blood samples were centrifuged, and the serum was separated and stored at −80 °C until analysis. Serum concentrations of IL-17A, PTGS2, and MMP9 were measured using commercially available human enzyme-linked immunosorbent assay (ELISA) kits according to the manufacturers’ instructions. Specifically, IL-17A (Ruixin Bio, RX106159H), PTGS2 (Ruixin Bio, RX2D182956), and MMP9 (Ruixin Bio, RX105770H) were quantified using their respective ELISA kits. All measurements were performed according to standardized laboratory procedures.

### 2.3 Animal experiments

#### 2.3.1 Animals and groups

Healthy adult male Sprague-Dawley rats, weighing 250–300 g, were purchased from Shanxi Sanxiangye Biotechnology Co., LTD. (License number: SCXK(Jin)2024-0003) and maintained in a standard SPF environment. Animals were housed under standard conditions (22 ± 2°C, 12-h light/dark cycle, with food and water ad libitum) for one week before the experiments. All animal experiments were approved by the Animal Ethics Committee of Shanxi Medical University (approval number: 2025077). For molecular analyses, rats were randomly assigned to three groups: sham-operated group, MCAO group, and IL-17A neutralizing antibody-treated MCAO group (n = 6 per group). Because TTC staining requires fresh brain tissue and precludes subsequent molecular assays in the same samples, an independent cohort of rats was used for infarct volume assessment. In this TTC cohort, 5 rats were included in the MCAO group and 5 rats in the IL-17A neutralizing antibody-treated group. One rat in the MCAO TTC cohort died before tissue collection, resulting in a final TTC sample size of n = 4 for the MCAO group and n = 5 for the IL-17A neutralizing antibody-treated group.

#### 2.3.2 Establishment of MCAO model in rats

The left middle cerebral artery occlusion model was established by the classical suture method (25). The brief procedure was as follows: rats were induced and maintained under anesthesia with 4% isoflurane and immobilized in the supine position. A median cervical incision was made, and the left common carotid artery, external carotid artery and internal carotid artery were isolated. The external carotid artery was ligated distal to the bifurcation of the common carotid artery, and the common carotid artery was temporarily blocked proximal with an arterial clip. A small incision was made in the common carotid artery, and a nylon wire plug coated with silicone at the tip was gently inserted through the internal carotid artery and stopped after slight resistance to block blood flow at the origin of the middle cerebral artery. After 90 min of ischemia, the wire plug was gently pulled out to achieve reperfusion. In the sham-operated group, all the other surgical procedures were the same except that the middle cerebral artery was not blocked. During recovery from anesthesia, the neurological deficits of rats were observed. Neurological deficits were evaluated 2 h after reperfusion using a 5-point scale (26). For MCAO-operated rats, animals with scores of 1–3 were included in the study, whereas rats with scores of 0 or 4 were excluded. Sham-operated rats were not subject to this neurological inclusion criterion. Separate animal cohorts were used for molecular analyses and TTC staining, with sample sizes detailed above.

#### 2.3.3 Drug intervention

Rats in the IL-17A neutralizing antibody-treated group were treated with Anti-Mouse/Rat IL-17A Antibody (17F3; MedChemExpress, Cat. No. HY-P990222), a mouse IgG1κ monoclonal neutralizing antibody against mouse/rat IL-17A, via intravenous injection at 100 µg/rat immediately after reperfusion and again at 12 h post-reperfusion. This dosing regimen was selected with reference to previous *in vivo* studies (27, 28) and the exploratory design of the present acute MCAO study, with the aim of blocking IL-17A activity during the early post-ischemic inflammatory phase. Rats in the sham and MCAO groups received an equal volume of PBS. No isotype-matched control IgG antibody was included in the present animal experiment; therefore, potential non-specific effects related to IgG administration could not be completely excluded.

#### 2.3.4 Neurological deficit assessment

Neurological deficits were evaluated at 24 h after reperfusion using the modified Longa 5-point scoring system by an investigator blinded to group allocation: 0, no neurological deficit; 1, failure to fully extend the right forepaw; 2, circling to the right; 3, falling to the right; and 4, inability to walk spontaneously with a depressed level of consciousness.

#### 2.3.5 TTC staining for infarct volume measurement

An independent cohort of rats was used for TTC staining and infarct volume analysis. At 24 h after reperfusion, rats were deeply anesthetized and the brains were rapidly removed and placed at −80 °C for 5 min. Coronal sections (2 mm thick) were cut using a brain matrix and incubated in 2% TTC solution (Sigma-Aldrich, USA) at 37 °C for 15 min in the dark, followed by fixation in 4% paraformaldehyde overnight. Infarcted tissue appeared white, whereas viable tissue appeared red. Images were captured using a digital camera, and infarct volume was quantified using ImageJ software. To correct for edema, corrected infarct volume (%) was calculated as (contralateral hemisphere area − ipsilateral non-infarcted area)/(contralateral hemisphere area) × 100%. In the TTC cohort, the final sample size was n = 4 for the MCAO group and n = 5 for the IL-17A neutralizing antibody-treated group because one rat in the MCAO group died before tissue collection.

#### 2.3.6 Tissue sample collection

Twenty-four hours after reperfusion, the rats were deeply anesthetized. Brains were harvested by rapid decapitation, and brain tissue from the left ischemic hemisphere was rapidly isolated on ice, loaded into cryopreservation tubes, immediately put into liquid nitrogen, and subsequently transferred to a −80 °C refrigerator for storage.

### 2.4 Molecular biological detection

#### 2.4.1 RNA extraction and RT-qPCR

Total RNA was extracted from frozen brain tissue using TRIzol reagent. cDNA was synthesized by reverse transcription using the SweScript All-in-One RT SuperMix for qPCR kit. Amplification reactions were performed on a Bio-Rad real-time PCR system using 2×Universal Blue SYBR Green qPCR Master Mix. Reaction conditions were as follows: initial denaturation at 95 °C for 30 s, followed by 40 cycles of 95 °C for 15 s and 60 °C for 30 s. GAPDH was used as the reference gene. The 2^(-ΔΔCt) method was used to calculate the relative gene expression. The primer sequences used for qPCR were as follows:

GAPDH: Forward, 5′-CTGGAGAAACCTGCCAAGTATG-3′; Reverse, 5′-GGTGGAAGAATGGGAGTTGCT-3′.

IL-17A: Forward, 5′-TCCTCTATTGTCCGCCATGC-3′; Reverse, 5′-ATTTGTATCCCCTCTGCGCC-3′.

PTGS2: Forward, 5′-CTCCTTGAACACGGACTTGCT-3′; Reverse, 5′-TAAGGTTTCAGGGAGAAGCGTT-3′.

MMP9: Forward, 5′-TCCAGCATCTGTATGGTCGTG-3′; Reverse, 5′-GCAGTGGGACACATAGTGGG-3′.

#### 2.4.2 ELISA detection

Frozen brain tissue was homogenized in precooled PBS at a defined weight-to-volume ratio on ice, followed by centrifugation at 10,000×g for 15 min at 4°C. The supernatant was collected for subsequent ELISA assays. Using commercial ELISA kits, the concentrations of IL-17A (MULTI SCIENCES, EK317/3), PTGS2 (Cloud-Clone Corp, SEA699Ra), MMP9 (Cloud-Clone Corp, SEA553Ra), PGE2 (Cloud-Clone Corp, MEA538Ge), and IL-6 (Invitrogen, 88-50625) in the supernatant were measured according to the manufacturer’s instructions. Protein concentrations were normalized using the BCA assay.

### 2.5 Statistical analysis

R software (v4.2.0) was used for statistical analysis. Data are presented as mean ± standard error of the mean (SEM) or as median with interquartile range, as appropriate. Data normality was assessed by the Shapiro-Wilk test and homogeneity of variance by the Levene test. One-way analysis of variance (ANOVA) followed by Tukey’s *post hoc* test was used for comparisons among multiple groups when data were normally distributed with homogeneous variance. Welch’s ANOVA followed by Games-Howell *post hoc* test was used when data were normally distributed but variances were unequal. Non-parametric data were analyzed using the Kruskal-Wallis test followed by Dunn’s multiple-comparison test. Independent-samples t test or Mann-Whitney U test was used for comparisons between two groups where appropriate. Pearson or Spearman correlation was used for correlation analysis as appropriate. No formal global correction across all analytes was applied; therefore, the possibility of type I error should be considered when interpreting the results. A two-sided P < 0.05 was considered statistically significant.

## 3 Results

### 3.1 Bioinformatics analysis prioritized PTGS2 and MMP9 as candidate inflammatory genes associated with the IL-17 signaling pathway

**Figure 1:**
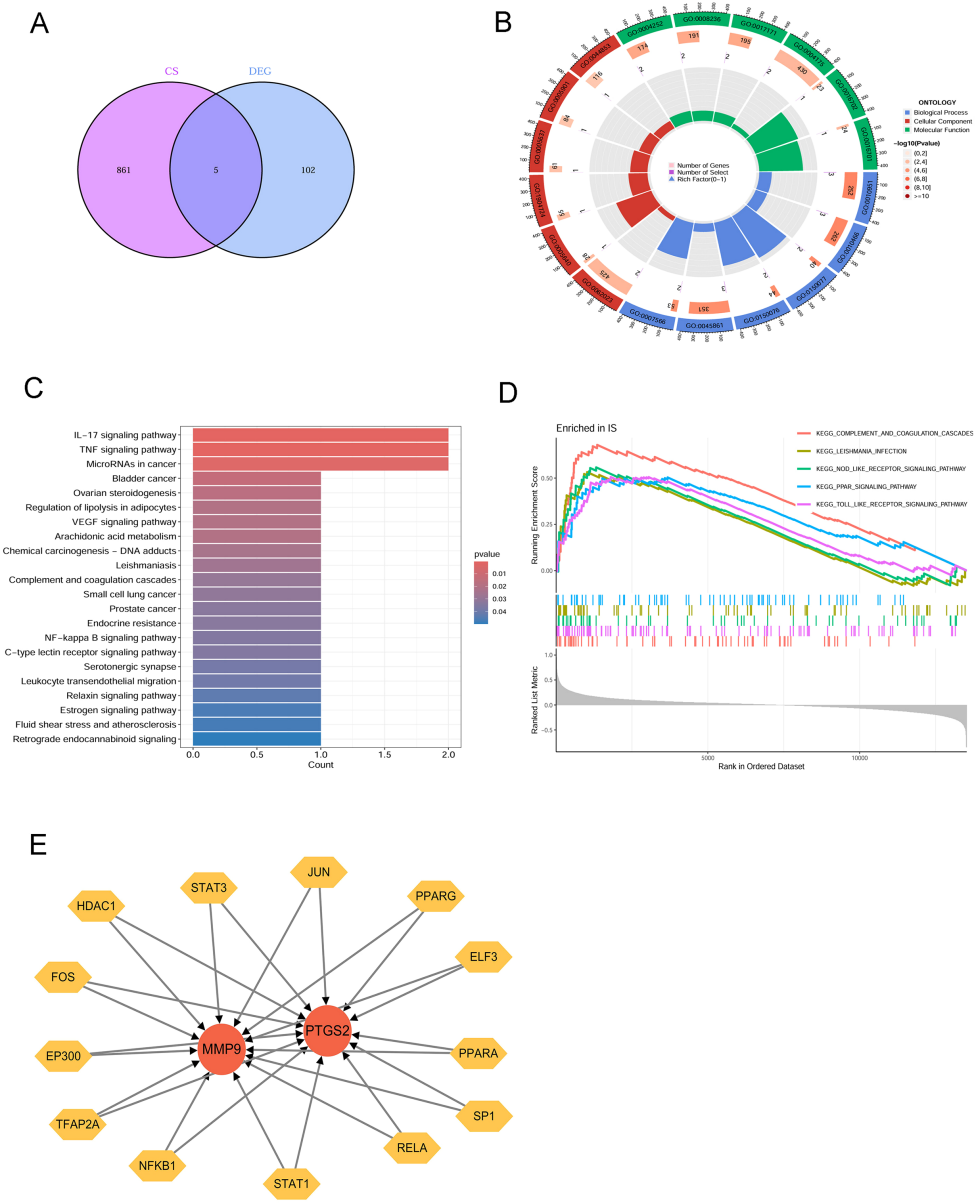
Bioinformatics analysis prioritized PTGS2 and MMP9 as candidate inflammatory genes associated with the IL-17 signaling pathway in ischemic stroke. **(A)** Venn diagram showing the overlap between differentially expressed genes (DEGs) identified from GEO datasets and genes retrieved from the CellAge database. Five overlapping genes were identified: PTGS2, MMP9, SERPINB2, CTNNAL1, and HTRA1. CS, genes from CellAge database; DEG, significantly differentially expressed genes in ischemic stroke. **(B)** Gene Ontology (GO) enrichment analysis of the overlapping genes, including biological process, cellular component, and molecular function categories. **(C)** Kyoto Encyclopedia of Genes and Genomes (KEGG) pathway enrichment analysis showing that the overlapping genes were enriched in inflammation-related pathways, including the IL-17 signaling pathway and TNF signaling pathway. **(D)** GSEA comparing ischemic stroke (IS) and control samples, demonstrating enrichment of immune- and inflammation-related pathways in IS. **(E)** Predicted transcription factor–target gene regulatory network for PTGS2 and MMP9, showing candidate shared upstream transcription factors. Yellow nodes represent TFs, red nodes represent target genes.

To identify candidate genes associated with ischemic stroke, we analyzed three GEO datasets of peripheral blood samples and screened differentially expressed genes showing consistent changes across datasets. Intersecting these ischemic stroke-related DEGs with CellAge-annotated genes yielded five overlapping genes—PTGS2, MMP9, SERPINB2, CTNNAL1, and HTRA1—which were retained for exploratory downstream analysis within an age-related biological context (**Figure 1A**).

GO enrichment analysis indicated that these overlapping genes were mainly involved in inflammatory and injury-related biological functions (**Figure 1B**). In the biological process category, the most enriched terms included neuroinflammatory response, regulation of neuroinflammatory response, and acute inflammatory response. In the molecular function category, serine-type endopeptidase activity and metalloendopeptidase activity were prominent, with MMP9 contributing to these annotations. Terms related to oxidoreductase activity and peroxidase activity were also enriched. In the cellular component category, the genes were primarily associated with the collagen-containing extracellular matrix and plasma membrane raft.

KEGG analysis showed that these genes were enriched in inflammation-related pathways, including the IL-17 signaling pathway and TNF signaling pathway (**Figure 1C**). Among them, PTGS2 and MMP9 were both annotated within the IL-17 signaling pathway, suggesting a potential association between IL-17-related inflammatory signaling and PTGS2/MMP9 expression in ischemic stroke.

GSEA comparing ischemic stroke and control samples further demonstrated enrichment of immune-related pathways in ischemic stroke, including Toll-like receptor signaling, NOD-like receptor signaling, complement and coagulation cascades, and PPAR signaling (**Figure 1D**), supporting the presence of systemic inflammatory and metabolic alterations.

To explore potential upstream regulatory mechanisms, transcription factor prediction analysis identified 13 candidate transcription factors that may co-regulate PTGS2 and MMP9 (**Figure 1E**), including RELA, NFKB1, STAT1, STAT3, JUN, FOS, PPARA, and PPARG. Collectively, these bioinformatics findings prioritized PTGS2 and MMP9 as candidate inflammatory genes linked to IL-17-associated signaling in ischemic stroke, while direct causal relationships require further experimental validation.

### 3.2 Baseline characteristics of the clinical cohort

The baseline characteristics of the study population are summarized in **Table 1**. A total of 104 patients with ischemic stroke and 58 control subjects were included in the analysis. Demographic variables, vascular risk factors, and relevant clinical characteristics, including age, sex, smoking history, alcohol consumption, diabetes, hypertension, leukoaraiosis, carotid plaque, admission NIHSS score, and time from symptom onset to blood sampling, were collected to characterize the clinical cohort and to facilitate interpretation of the biomarker findings.

**Table 1:** Baseline characteristics of patients with ischemic stroke and control subjects.

Characteristic	IS (n = 104)	Control (n = 58)	P value
Age, years	67.00 (62.50, 69.00)	64.00 (61.00, 68.00)	0.180
Sex, n (%)			0.402
Female	36 (35%)	24 (41%)	
Male	68 (65%)	34 (59%)	
Smoking history, n (%)			<0.001
No	58 (56%)	52 (90%)	
Yes	46 (44%)	6 (10%)	
Alcohol consumption, n (%)			0.006
No	81 (78%)	55 (95%)	
Yes	23 (22%)	3 (5.2%)	
Diabetes mellitus, n (%)			0.244
No	77 (74%)	48 (83%)	
Yes	27 (26%)	10 (17%)	
Hypertension, n (%)			<0.001
No	25 (24%)	33 (57%)	
Yes	79 (76%)	25 (43%)	
Coronary heart disease, n (%)			0.497
No	96 (92%)	56 (97%)	
Yes	8 (7.7%)	2 (3.4%)	
Leukoaraiosis, n (%)			<0.001
No	52 (50%)	45 (78%)	
Yes	52 (50%)	13 (22%)	
Carotid plaque, n (%)			<0.001
No	19 (18%)	40 (69%)	
Yes	85 (82%)	18 (31%)	
Triglycerides, mmol/L	1.06 (0.88, 1.64)	1.43 (0.94, 1.88)	0.091
Total cholesterol, mmol/L	3.87 (3.37, 4.38)	4.16 (3.67, 4.62)	0.133
LDL-C, mmol/L	2.38 (1.99, 2.89)	2.70 (2.20, 2.98)	0.165
HDL-C, mmol/L	1.00 (0.84, 1.23)	1.00 (0.88, 1.21)	0.803
Homocysteine, μmol/L	17.30 (12.15, 22.85)	15.45 (10.70, 21.80)	0.262
Fasting blood glucose, mmol/L	5.10 (4.59, 5.88)	5.31 (4.66, 6.23)	0.248
HbA1c, %	5.60 (5.30, 6.07)	5.80 (5.50, 6.60)	0.038
D-dimer, mg/L	0.51 (0.40, 0.80)	0.50 (0.40, 0.75)	0.466
Fibrinogen, g/L	2.61 (2.22, 3.10)	2.73 (2.20, 3.26)	0.675
NIHSS score at admission	3.00 (2.00, 6.00)	NA	NA
Time from symptom onset to blood sampling, h	9.00 (7.00, 12.00)	NA	NA
TOAST classification, n (%)		NA	NA
Large-artery atherosclerosis	42 (40%)		
Cardioembolism	11 (11%)		
Small artery occlusion	48 (46%)		
Other determined etiology and undetermined etiology	3 (2.9%)		
Stroke territory, n (%)		NA	NA
Posterior circulation	33 (32%)		
Anterior circulation	71 (68%)		

Data are presented as median (Q1, Q3), or n (%), as appropriate. Between−group comparisons were performed using the Mann–Whitney U test for continuous variables and the χ² test or Fisher’s exact test for categorical variables. Other determined etiology and undetermined etiology were combined because of the small number of cases. IS, ischemic stroke; NIHSS, National Institutes of Health Stroke Scale; LDL−C, low−density lipoprotein cholesterol; HDL−C, high−density lipoprotein cholesterol; HbA1c, glycated hemoglobin; NA, not applicable.

### 3.3 Association of ischemic stroke status with serum IL-17A, PTGS2, and MMP9 levels in the clinical cohort

**Figure 2:**
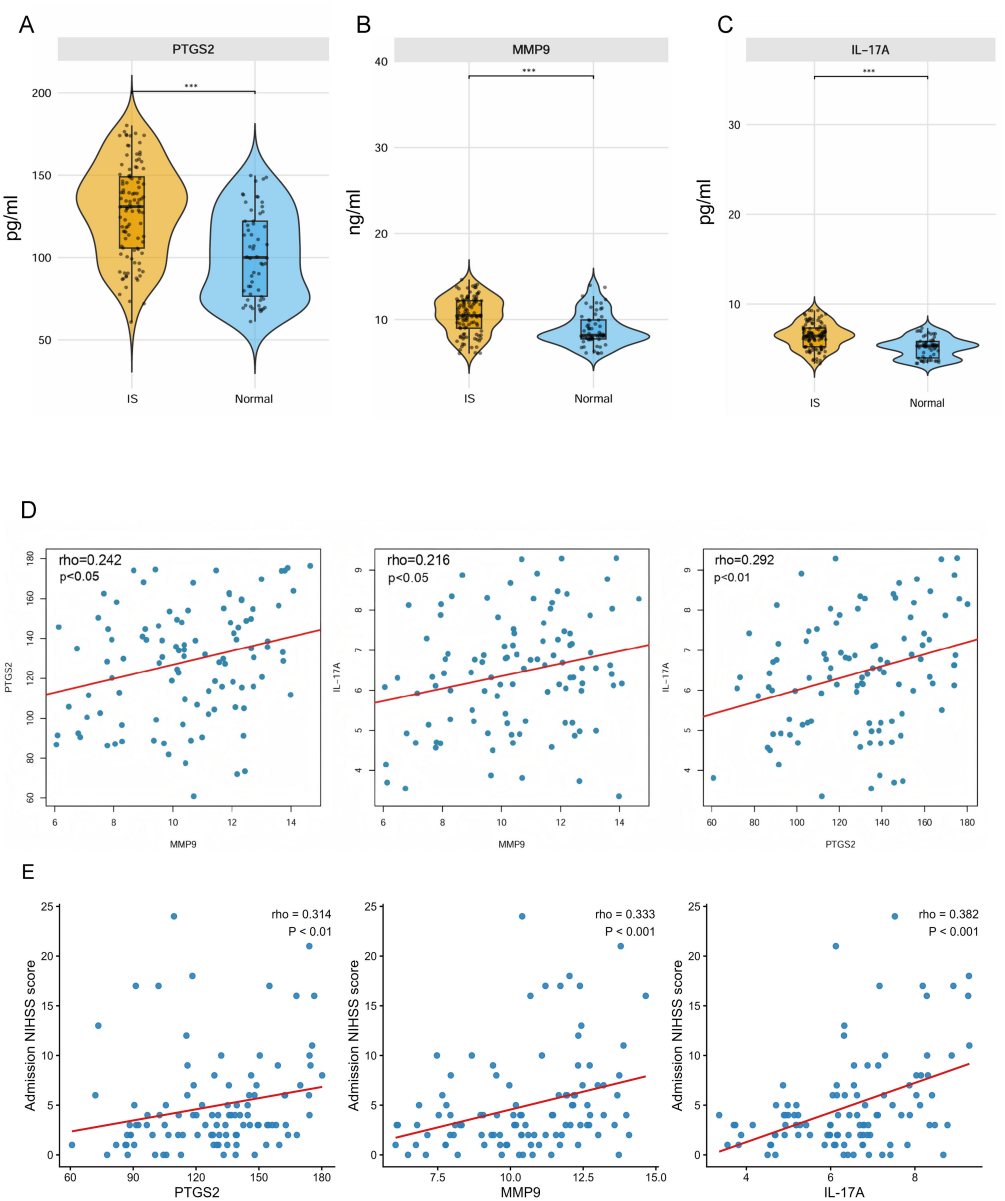
Serum IL-17A, PTGS2, and MMP9 levels in the clinical cohort and their correlations with each other and with stroke severity. **(A–C)** Serum IL-17A, PTGS2, and MMP9 levels were significantly higher in patients with ischemic stroke than in healthy controls. IS, ischemic stroke (n = 104); Normal, control group (n = 58). **(D)** Correlations among serum IL-17A, PTGS2, and MMP9 levels in the stroke group. **(E)** Positive correlations between serum IL-17A, PTGS2, and MMP9 levels and admission NIHSS scores in patients with ischemic stroke. Correlations were analyzed using Spearman’s rank correlation test. *P < 0.05, **P < 0.01, ***P < 0.001.

Serum IL-17A, PTGS2, and MMP9 levels were significantly elevated in patients with ischemic stroke compared with healthy controls (all P < 0.001; **Figures 2A–C**). To assess whether these differences were independent of baseline covariates, we performed multivariable linear regression analyses with each biomarker as the dependent variable and ischemic stroke status as the primary independent variable. After adjustment for age, sex, smoking history, alcohol consumption, diabetes, and hypertension, ischemic stroke remained significantly associated with higher serum IL-17A, PTGS2, and MMP9 levels. These associations were materially unchanged in a sensitivity model further adjusted for leukoaraiosis and carotid plaque. In the fully adjusted model, ischemic stroke was independently associated with higher IL-17A (β = 0.20, 95% CI 0.11–0.30, P < 0.001), PTGS2 (β = 0.19, 95% CI 0.09–0.30, P < 0.001), and MMP9 (β = 0.19, 95% CI 0.10–0.27, P < 0.001) levels (**Table 2**).

**Table 2:** Multivariable linear regression analysis of the association between ischemic stroke and serum IL-17A, PTGS2, and MMP9 levels.

Biomarker	β (95% CI), M1	β (95% CI), M2	β (95% CI), M3	P, M1	P, M2	P, M3
IL-17A	0.22 (0.15, 0.30)	0.24 (0.15, 0.32)	0.20 (0.11, 0.30)	<0.001	<0.001	<0.001
PTGS2	0.24 (0.16, 0.32)	0.22 (0.13, 0.31)	0.19 (0.09, 0.30)	<0.001	<0.001	<0.001
MMP9	0.16 (0.09, 0.23)	0.17 (0.09, 0.25)	0.19 (0.10, 0.27)	<0.001	<0.001	<0.001

Multivariable linear regression models were constructed with serum biomarker levels as dependent variables and ischemic stroke status as the primary independent variable. Model 1 was unadjusted. Model 2 was adjusted for age, sex, smoking history, alcohol consumption, diabetes, and hypertension. Model 3 was further adjusted for leukoaraiosis and carotid plaque. Biomarker levels were log-transformed before linear regression analysis. β, the adjusted regression coefficient for ischemic stroke status; CI, confidence interval; M1, Model 1; M2, Model 2; M3, Model 3.

Within the stroke group, Spearman correlation analysis showed that serum IL-17A was positively correlated with PTGS2 (rho = 0.292, P < 0.01) and MMP9 (rho = 0.216, P < 0.05), while PTGS2 was also positively correlated with MMP9 (rho = 0.242, P < 0.05) (**Figure 2D**). Together, these findings support a coordinated inflammatory relationship among IL-17A, PTGS2, and MMP9 in ischemic stroke.

### 3.4 Association of serum IL-17A, PTGS2, and MMP9 levels with clinical severity in patients with ischemic stroke

To further assess the clinical relevance of the IL-17A/PTGS2-MMP9 axis, we examined the relationship between serum biomarker levels and admission NIHSS score in the stroke cohort. Spearman correlation analysis showed that serum IL-17A levels were positively correlated with NIHSS score (rho = 0.382, P < 0.001), and similar positive correlations were also observed for PTGS2 (rho = 0.314, P < 0.01) and MMP9 (rho = 0.333, P < 0.001) (**Figure 2E**). These findings indicate that higher levels of this inflammatory axis are associated with more severe neurological impairment at presentation.

To further determine whether these associations were independent of potential confounding factors, we performed multivariable linear regression analyses with admission NIHSS score as the dependent variable and log-transformed biomarker levels as the main independent variables. After adjustment for age, sex, time from symptom onset to blood sampling, diabetes, and hypertension, higher levels of all three biomarkers remained independently associated with higher admission NIHSS score. Specifically, log-transformed IL-17A (β = 8.49, 95% CI 4.99–11.99, P < 0.001), PTGS2 (β = 3.99, 95% CI 0.30–7.67, P = 0.034), and MMP9 (β = 7.44, 95% CI 3.45–11.44, P < 0.001) were each significantly associated with greater neurological deficit at admission (**Table 3**). Together, these results indicate that elevated serum IL-17A, PTGS2, and MMP9 levels are associated not only with the presence of ischemic stroke, but also with greater clinical severity.

**Table 3:** Multivariable linear regression analysis of the association between serum IL-17A, PTGS2, and MMP9 levels and admission NIHSS score in patients with ischemic stroke.

Biomarker	β (95% CI)	P value
IL-17A	8.49 (4.99-11.99)	<0.001
PTGS2	3.99 (0.30-7.67)	0.034
MMP9	7.44 (3.45-11.44)	<0.001

Separate multivariable linear regression models were constructed with admission NIHSS score as the dependent variable and each log-transformed serum biomarker as the primary independent variable. Models were adjusted for age, sex, time from symptom onset to blood sampling, diabetes, and hypertension.

### 3.5 MCAO activated IL-17A/PTGS2/MMP9-related inflammatory signaling in rat brain tissue

**Figure 3:**
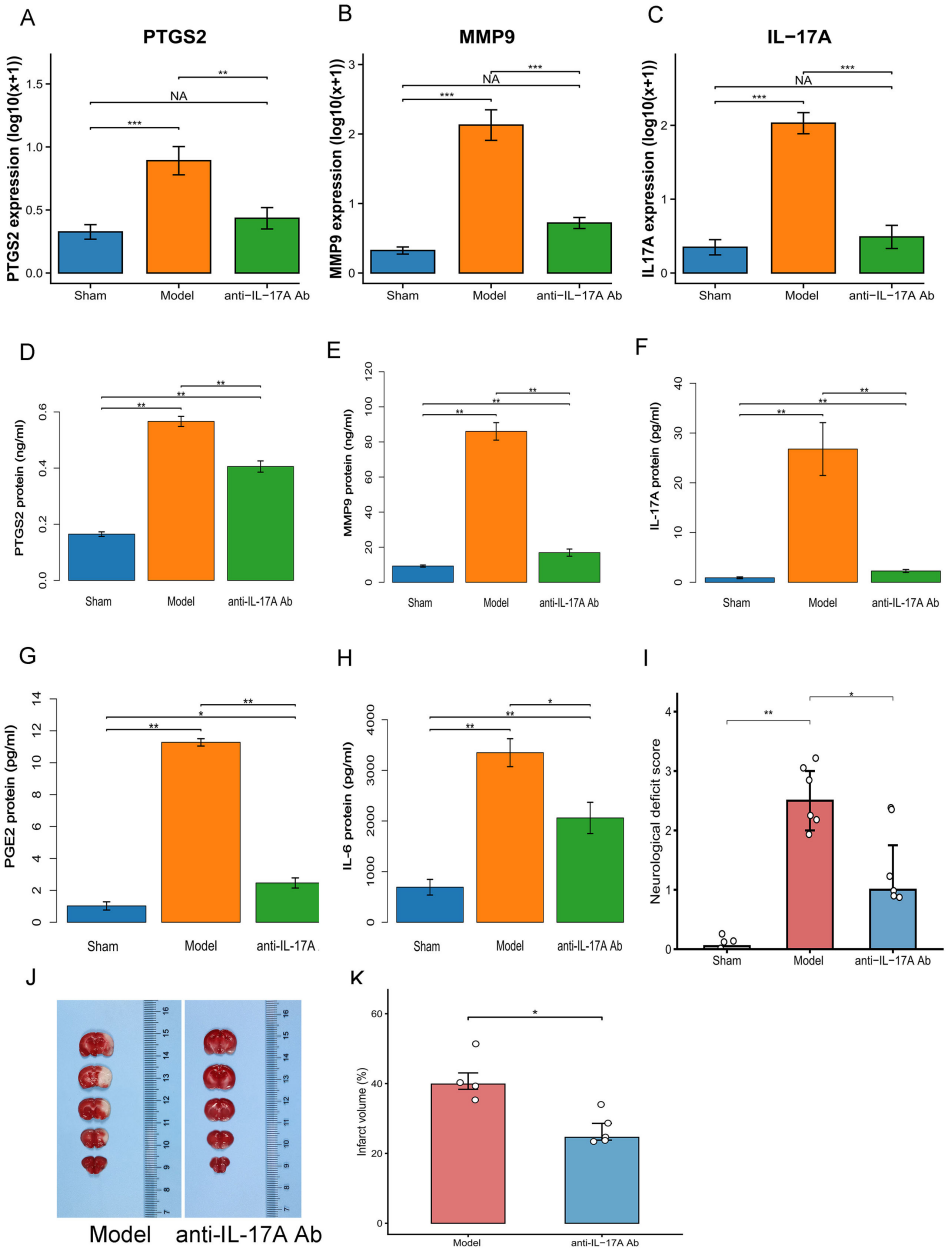
MCAO-induced activation of IL-17A/PTGS2/MMP9-related inflammatory markers and changes after anti-IL-17A antibody treatment in rat brain tissue. **(A–C)** Relative mRNA levels of PTGS2, MMP9, and IL-17A in the sham, MCAO, and anti-IL-17A Ab groups. **(D–F)** Protein concentrations of PTGS2, MMP9, and IL-17A. **(G, H)** Concentrations of PGE2 and IL-6. **(I)** Neurological deficit scores assessed using the Longa scoring system. In panel I, the sham group median was 0; therefore, a minimal bar height was displayed for visualization only. **(J)** Representative TTC-stained brain sections from the MCAO and anti-IL-17A Ab groups. White areas indicate infarct regions, whereas red areas indicate viable brain tissue. **(K)** Quantitative analysis of infarct volume percentage based on TTC staining. Sham, sham-operated group; Model, MCAO group; anti-IL-17A Ab, IL-17A neutralizing antibody-treated MCAO group. Data in panels A-H are presented as mean ± SEM (n = 6 per group). Neurological deficit scores in panel I are presented as median (interquartile range) (n = 6 per group). TTC analysis in panels **(J, K)** was performed in an independent cohort of rats, with final sample sizes of n = 4 for the MCAO group and n = 5 for the anti-IL-17A Ab group; one rat in the MCAO group died before tissue collection. *P < 0.05, **P < 0.01, ***P < 0.001.

To determine whether the IL-17A/PTGS2/MMP9 axis was activated after cerebral ischemia-reperfusion injury, we measured IL-17A, PTGS2, and MMP9 in brain tissue from sham-operated and MCAO rats. Compared with the sham group, MCAO markedly increased both mRNA and protein levels of IL-17A. Consistent with this increase, PTGS2 expression and its downstream product PGE2 were also significantly elevated in the MCAO group. MMP9 expression was likewise significantly increased, accompanied by higher IL-6 levels (**Figures 3A–H**). These findings indicate that cerebral ischemia-reperfusion injury is associated with activation of IL-17A-related inflammatory signaling together with upregulation of PTGS2, MMP9, and related downstream inflammatory mediators *in vivo*.

### 3.6 IL-17A neutralizing antibody treatment was associated with attenuated inflammatory signaling and improved ischemic outcomes after MCAO

To further investigate the *in vivo* role of IL-17A, rats subjected to MCAO were treated with an IL-17A neutralizing antibody. Compared with the PBS-treated MCAO group, IL-17A neutralization significantly reduced IL-17A levels in brain tissue at both the mRNA and protein levels. In parallel, PTGS2 and MMP9 mRNA expression was significantly decreased in the intervention group. ELISA further showed reduced protein levels of PTGS2 and MMP9, together with lower concentrations of PGE2 and IL-6 (**Figures 3A–H**). These results suggest that IL-17A neutralization is associated with attenuation of PTGS2/PGE2- and MMP9-related inflammatory changes after cerebral ischemia-reperfusion injury. However, because an isotype-matched IgG control was not included, potential non-specific effects of antibody administration cannot be fully excluded and the intervention results should be interpreted cautiously.

We next assessed whether these molecular changes were accompanied by improvement in ischemic injury severity. Neurological deficits were evaluated using the Longa scoring system in sham-operated, MCAO, and IL-17A neutralizing antibody-treated rats. As expected, rats in the MCAO group exhibited significantly worse neurological deficits than those in the sham group. Importantly, neurological deficit scores were significantly lower in the IL-17A neutralizing antibody-treated group than in the MCAO group [1.00 (1.00-1.75) vs. 2.50 (2.00-3.00), P < 0.05], indicating improved neurological outcomes after anti-IL-17A antibody treatment. Cerebral infarct volume was further assessed by TTC staining in an independent cohort of rats. The MCAO group showed substantial infarction, whereas treatment with IL-17A neutralizing antibody significantly reduced infarct volume [24.60% (23.80-28.60%) vs. 39.85% (38.35-43.05%), P < 0.05] (**Figures 3J, K**). Together, these findings suggest that anti-IL-17A antibody treatment was associated with attenuation of IL-17A/PTGS2/MMP9-related inflammatory signaling, reduced ischemic injury indices, and improved neurological outcomes in this model; however, these results should be interpreted cautiously because an isotype-matched IgG control was not included.

**Figure 4:**
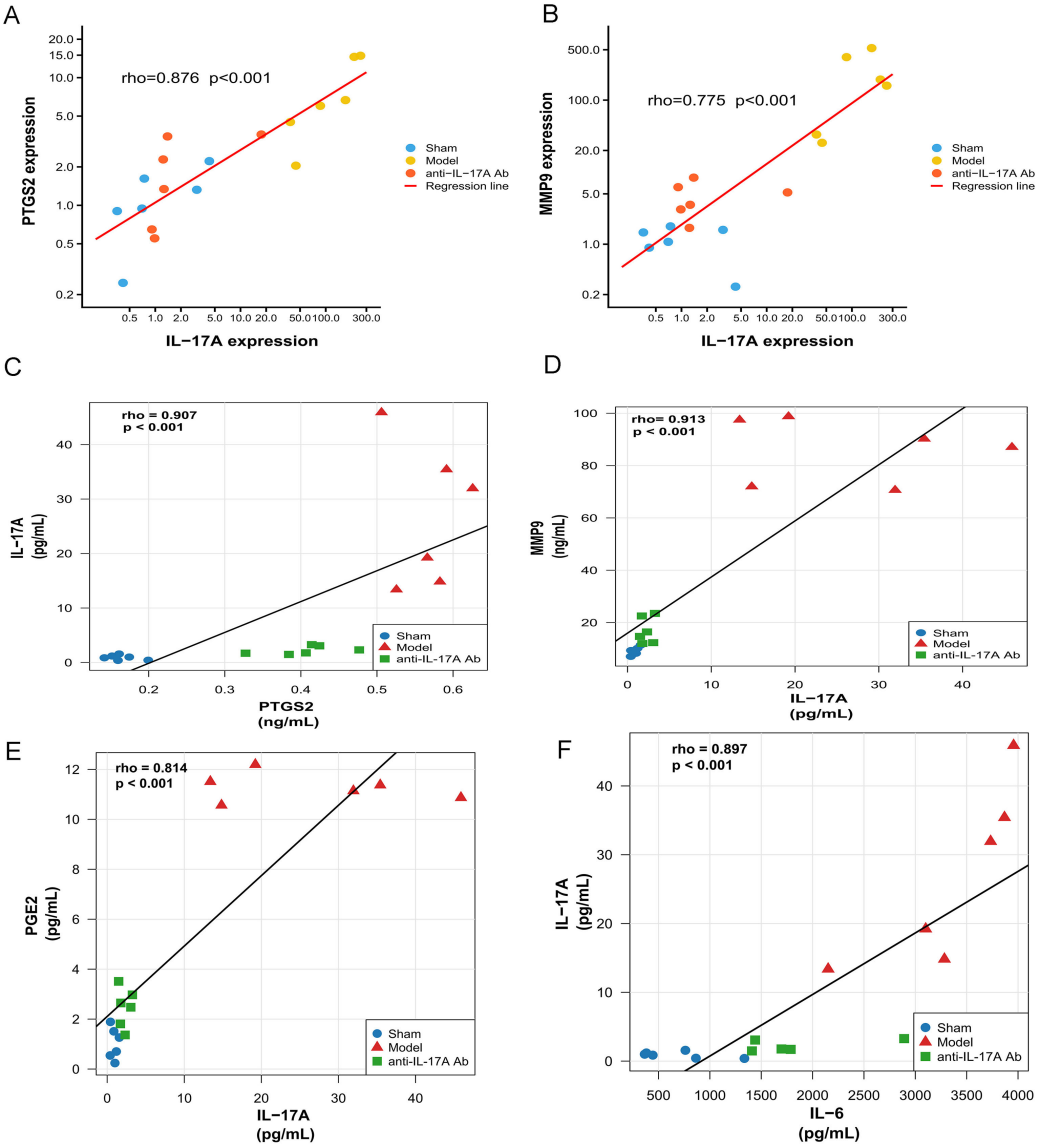
Spearman correlation analyses among IL-17A, PTGS2, MMP9, PGE2, and IL-6 in rat brain tissue. Correlation analyses were performed using pooled data from all animals (n = 18). **(A)** Correlation between IL-17A and PTGS2 mRNA expression. **(B)** Correlation between IL-17A and MMP9 mRNA expression. **(C)** Correlation between IL-17A and PTGS2 protein expression. **(D)** Correlation between IL-17A and MMP9 protein expression. **(E)** Correlation between IL-17A and PGE2 levels. **(F)** Correlation between IL-17A and IL-6 levels. Sham, sham-operated group; Model, MCAO group; anti-IL-17A Ab, IL-17A neutralizing antibody-treated MCAO group. Given the limited sample size, these correlations should be interpreted with caution.

When data from all animals were pooled, Spearman correlation analysis further showed coordinated positive associations among IL-17A, PTGS2, MMP9, PGE2, and IL-6 (**Figure 4**). In particular, IL-17A was positively correlated with PTGS2 and MMP9 at both the mRNA and protein levels, and IL-17A protein levels were also positively correlated with PGE2 and IL-6. Although these findings further support a coordinated inflammatory relationship among these molecules in the MCAO model, these correlations should be interpreted cautiously given the limited sample size.

## 4 Discussion

In the present study, we combined bioinformatics analysis, clinical validation, and experimental MCAO data to investigate the potential role of IL-17A-associated inflammatory signaling in ischemic stroke. Our main findings were that PTGS2 and MMP9 emerged as inflammation-related candidate genes linked to IL-17 signaling, that serum IL-17A, PTGS2, and MMP9 were elevated in patients with ischemic stroke and associated with stroke severity, and that anti-IL-17A antibody treatment in MCAO rats was associated with attenuated PTGS2/MMP9-related inflammatory responses, smaller infarct volume, and improved neurological deficit scores.

The integrated bioinformatics analyses strengthen the biological plausibility of this proposed inflammatory network. PTGS2 and MMP9 were enriched in neuroinflammatory and acute inflammatory processes and were annotated within the IL-17 and TNF signaling pathways. GSEA further indicated activation of innate immune and vascular-inflammatory programs, including Toll-like receptor signaling, NOD-like receptor signaling, and complement/coagulation cascades, while transcription factor analysis identified shared regulators such as RELA, NFKB1, and STAT3. Together, these pathway-level findings suggest that IL-17A-associated signaling may participate in a broader inflammatory amplification network rather than acting as an isolated pathway. These systems-level findings were supported by the clinical data, in which circulating IL-17A, PTGS2, and MMP9 were all elevated in ischemic stroke and remained independently associated with stroke status after multivariable adjustment. Their additional association with admission NIHSS score further suggests that this inflammatory signature is linked not only to disease presence but also to clinical severity.

The animal experiments extended these observations from clinical association to preclinical intervention. Cerebral ischemia/reperfusion was accompanied by increased IL-17A expression in brain tissue together with elevated PTGS2, PGE2, MMP9, and IL-6, indicating activation of a broad inflammatory response after MCAO. Importantly, IL-17A neutralizing antibody treatment was associated with reductions in these molecular changes, suggesting that IL-17A appears to contribute to PTGS2/PGE2- and MMP9-related inflammatory responses in the ischemic brain. These findings are consistent with prior evidence that IL-17A is involved in post-ischemic inflammation, and in our study they place PTGS2 and MMP9 within an IL-17A-associated inflammatory context.

Nevertheless, the interpretation of the antibody intervention experiment requires caution. In the present study, PBS rather than an isotype-matched non-specific IgG antibody was used as the control for the IL-17A neutralizing antibody. Therefore, although anti-IL-17A antibody treatment was associated with reduced PTGS2/MMP9-related inflammatory responses, smaller infarct volume, and improved neurological deficit scores, we cannot completely exclude the possibility that non-specific IgG-related immune effects may have contributed to the observed differences. Accordingly, these findings should be interpreted as supportive evidence for the involvement of IL-17A-associated inflammatory signaling rather than definitive proof of antibody-specific therapeutic efficacy. Future studies including an isotype-matched IgG control are required to validate the specificity of IL-17A blockade in this model.

An additional point that warrants consideration is the likely cellular origin of the observed molecular changes. Because the present analyses were performed in whole brain homogenates, the specific cellular sources of IL-17A, PTGS2, and MMP9 could not be resolved. In the setting of ischemic stroke, IL-17A has been reported to arise predominantly from infiltrating immune populations, particularly Th17 cells and γδ T cells (29), although contributions from other inflammatory cells cannot be excluded. By contrast, PTGS2 and MMP9 likely reflect multicellular inflammatory responses involving both CNS-resident and infiltrating cells. PTGS2 may be induced in microglia/macrophages, astrocytes, endothelial cells, and sometimes neurons (30), whereas MMP9 is commonly linked to neutrophils, microglia/macrophages, and vascular-associated cells (31). Therefore, the reductions observed after IL-17A neutralization probably represent an integrated tissue-level response rather than modulation of a single cell type. Future studies using immunofluorescence co-localization, flow cytometry, or single-cell transcriptomic approaches will be necessary to clarify the cell-specific organization of this response.

Beyond molecular changes, the study also demonstrated functional relevance *in vivo*. IL-17A neutralizing antibody treatment improved neurological deficit scores and reduced infarct volume after MCAO, indicating that the observed anti-inflammatory effects were accompanied by meaningful protection against ischemic brain injury. Mechanistically, these findings are compatible with a model in which PTGS2/PGE2 reflects inflammatory mediator amplification (32), whereas MMP9 more directly relates to extracellular matrix degradation and neurovascular injury (33). However, because BBB permeability and brain edema were not directly assessed, conclusions regarding BBB preservation should remain cautious.

Several limitations should be acknowledged. First, although IL-17A neutralization attenuated PTGS2/MMP9-related inflammatory changes, the absence of exogenous IL-17A stimulation experiments limits conclusions regarding direct causality. Second, the study focused on IL-17A and did not systematically assess other IL-17 family ligands. Third, several limitations exist in the animal experiments. BBB integrity and brain edema were not directly measured, and only a single 24 h reperfusion time point was examined. Importantly, PBS rather than an isotype-matched non-specific IgG antibody was used as the control for the IL-17A neutralizing antibody. Therefore, although IL-17A neutralizing antibody treatment was associated with reduced inflammatory marker expression, smaller infarct volume, and improved neurological deficits, we cannot fully exclude potential non-specific effects related to IgG administration or IgG-associated immune responses. Future studies using an isotype-matched IgG control, as well as dose-response and time-course designs, are needed to confirm the specificity and robustness of IL-17A blockade in ischemic stroke. Fourth, the clinical cohort was single-center and cross-sectional, which limits causal inference despite multivariable adjustment. Finally, CellAge was used only as an exploratory gene-prioritization tool because ischemic stroke is closely associated with aging and aging/senescence-related processes may influence post-ischemic inflammation and vascular injury. However, CellAge is not a dedicated inflammation-related gene database, and cellular senescence was not directly assessed in the present study. Therefore, the current findings should not be interpreted as establishing a senescent phenotype or an immune-senescence axis. Instead, CellAge-based screening was used to narrow the candidate gene list within an age-related biological context. Future studies with ligand-specific comparisons, direct stimulation experiments, longitudinal designs, and larger prospective cohorts are warranted.

## 5 Conclusion

In conclusion, our findings support an association between IL-17A-centered inflammatory signaling and ischemic stroke across bioinformatic, clinical, and experimental levels. PTGS2 and MMP9 emerged as candidate inflammatory mediators within this network, and IL-17A neutralizing antibody treatment was associated with reduced inflammatory responses and improved ischemic injury indices in MCAO rats. However, because an isotype-matched IgG control was not included in the animal intervention experiment, the specificity of the antibody-mediated effect requires further validation. These results indicate that IL-17A-associated inflammation may be a biologically relevant pathway in ischemic stroke, but additional studies with appropriate antibody controls are needed before therapeutic conclusions can be drawn.

## Data Availability

The original contributions presented in the study are included in the article/supplementary material. Further inquiries can be directed to the corresponding author/s.
